# Recent advances in non-surgical management of cancer in the elderly

**DOI:** 10.12688/f1000research.14264.1

**Published:** 2018-11-15

**Authors:** Emily J. Guerard, Gil E. Harmon, Kieran D. Sahasrabudhe, Noelle K. LoConte

**Affiliations:** 1 Department of Medicine, University of Wisconsin School of Medicine and Public Health, 600 Highland Avenue, Madison, WI 53792, USA; 2 University of Wisconsin Carbone Cancer Center, 600 Highland Avenue, Madison, WI 53792, USA, USA

**Keywords:** geriatric assessment, oncology, chemotherapy, elderly, palliative care

## Abstract

This article summarizes the seminal publications from mid-2016 through 2017 in the area of medical care for older adults with cancer. Areas addressed include chemotherapy tolerance and efficacy in the aged, geriatric fitness assessments, and advancements in palliative and supportive care. The practice-changing finding from this past year’s publications is that antipsychotics should not be used in the management of terminal delirium in older adults receiving palliative care. The other trials demonstrated an improved understanding of the utility of geriatric assessments in patients with cancer, developed the body of information about which chemotherapy agents are safe and effective in older adults (and which are not), and expanded our understanding of good palliative and supportive care.

## Introduction

The population continues to “gray” as the baby boomers reach retirement age and beyond. The 2010 US census found a 15% increase in adults 65 years and older when compared with the 2000 US census. Moreover, the population that was 45 to 64 years old increased by 31.5%. One of the biggest risk factors for most cancers continues to be age and therefore the incidence of cancer is expected to continue to increase dramatically. Thus, incorporating optimal ways of caring for older patients with cancer is a critical skill for all oncologists. This review will include the significant publications in the area of care for older adults with cancer from mid-2016 to 2017. Areas addressed include chemotherapy tolerance and efficacy in the aged, geriatric fitness assessments, and advancements in palliative and supportive care.

## Chemotherapy tolerance in the aged

The first study, by Huerter *et al*. ^1^, sought to fill in the gap of how older adults with advanced non-small cell lung cancer (NSCLC) tolerate combination chemotherapy. The authors conducted a single-arm phase II study of vinorelbine and paclitaxel in adults over 70 years old with advanced NSCLC. The current standard of care for fit elderly patients with advanced NSCLC is a carboplatin-based doublet chemotherapy regimen. This was informed by a trial by Quoix *et al*. ^2^ in 2011 in which the combination of carboplatin and paclitaxel was compared with single-agent vinorelbine and single-agent gemcitabine for adults 70 to 89 years of age with advanced NSCLC and an Eastern Cooperative Oncology Group (ECOG) performance status (PS) of 0 to 2. Vinorelbine and gemcitabine were both considered acceptable monotherapy options for this patient population at the time. The trial found that the carboplatin–paclitaxel combination was superior to monotherapy in terms of median progression-free survival (PFS), median overall survival (OS), and response rate. However, the combination therapy was also associated with a higher rate of adverse events, including grade 3 or 4 neutropenia, febrile neutropenia, anemia, thrombocytopenia, and sensory neuropathy. Huerter *et al*. cite another study, by Belani and Fossella ^3^, which was a subgroup analysis by age of the TAX 326 trial comparing docetaxel–cisplatin, docetaxel–carboplatin, and vinorelbine–cisplatin in the treatment of advanced NSCLC. This analysis found that patients who were at least 65 years old experienced moderately higher rates of grade 3 or 4 asthenia, infection, and lung toxicities across treatment arms and diarrhea and sensory neurotoxicity for cisplatin‐containing arms compared with patients younger than 65. Given concerns regarding the toxicity of platinum-containing combination regimens in elderly patients, the goal of the trial by Huerter *et al*. was to determine whether the combination of vinorelbine and paclitaxel would demonstrate efficacy while producing less toxicity. The majority of patients enrolled had an excellent ECOG PS. This trial unfortunately showed a high rate of toxicity; 6 out of 19 patients (31.5%) developed grade 4 or 5 non-hematologic toxicity. The high rates of toxicity observed make this an unacceptable regimen for older adults with NSCLC.

A subgroup analysis of the AURELIA trial of bevacizumab added to chemotherapy for platinum-resistant ovarian cancer was also published in 2017 ^4^. This post-hoc analysis looked at the safety and efficacy of the addition of bevacizumab in patients over the age of 65 years relative to younger patients. The magnitude of PFS improvement from bevacizumab was the same for both older and younger patients (hazard ratios [HRs] of 0.49 versus 0.44, respectively, *p* = 0.58). Bevacizumab did add a significant benefit to both PFS and response rate but not to OS. The OS finding is thought to possibly be due to a high rate of crossover between the arms. Older adults did experience more grade 3 (or higher) hypertension with bevacizumab. However, no other toxicity, including thromboembolic events, was more common in older adults. This study suggests that bevacizumab is well tolerated in fit older females with platinum-refractory ovarian cancer.

Immune checkpoint inhibitors are being increasingly used in the treatment of multiple cancer types. This includes the use of nivolumab and pembrolizumab which target the programmed death-1/programmed death-1 ligand (PD-1/PD-1L) pathway for the treatment of advanced NSCLC. A review by Carmichael *et al*. ^5^ discusses trials studying these checkpoint inhibitors for the treatment of advanced NSCLC that include elderly patients and gives information regarding subgroup analyses by age. One of the included studies is a trial by Reck *et al*. ^6^ of 305 patients with previously untreated advanced NSCLC with PD-1L expression on at least 50% of tumor cells who, owing to a lack of a sensitizing mutation in the epidermal growth factor receptor gene or translocation in the anaplastic lymphoma kinase gene, were not candidates for targeted therapy. Participants were randomly assigned to receive treatment with either pembrolizumab or platinum-based chemotherapy. Median PFS rates were 10.3 months in the pembrolizumab group and 6.0 months in the chemotherapy group (HR for disease progression or death, 0.50; 95% confidence interval [CI] 0.37 to 0.68; *p* <0.001). A subgroup analysis by age shows that this PFS benefit existed in the 141 patients younger than 65 years old (HR 0.61, 95% CI 0.40–0.92) and in the 164 patients who were 65 and older (HR 0.45, 95% CI 0.29–0.70), and this suggests that pembrolizumab is associated with a PFS advantage in elderly patients as well as younger patients. However, it should be noted that other studies involving subgroup analyses with age cutoffs of 65 and 75 show a trend toward no benefit for checkpoint inhibitors in patients older than 75. This needs to be considered when interpreting the results of the trial by Reck *et al*. given that the cutoff for the elderly subgroup was 65 years and that separate data are not reported for higher age cutoffs. Additional clinical trials focusing on patients over the age of 75 will be needed in order to develop a better understanding of the efficacy of checkpoint inhibitors for the treatment of advanced NSCLC in this patient population. Another study included in the review article is an abstract by Spigel *et al*. ^7^, who conducted subgroup analyses by age and PS by using data from the ongoing CheckMate 153 trial. This is a phase 3B/4 safety study of nivolumab in patients with metastatic NSCLC who have failed prior treatment. The subgroup analysis separated results for 788 patients under the age of 70 and 520 patients 70 and older. The authors found similar rates of toxicity in each group; the percentages of patients experiencing grade 3 to 4 treatment-related adverse events were 11% for patients under 70 and 13% for patients 70 or older. This suggests that older adults tolerate treatment similarly to younger patients. More clinical trials involving elderly patients will be needed in order to further assess the efficacy and safety of immune checkpoint inhibitors in this population.

Another important area of study in geriatric oncology consists of late effects associated with cancer-directed therapies. The incidence of prostate cancer increases with age, and patients often live for many years even after being diagnosed with metastatic disease. Understanding the potential long-term adverse health effects associated with therapy for prostate cancer is therefore important. A study by Hershman *et al*. ^8^ addresses long-term health effects associated with androgen deprivation therapy (ADT) for the treatment of metastatic prostate cancer by using data from patients who had participated in the SWOG S9346 study ^9^. The SWOG S9346 trial compared continuous versus intermittent ADT in the treatment of metastatic prostate cancer. Patients were followed for 10 years after initial registration or until death, whichever came first. The results of the study were inconclusive with regard to whether intermittent therapy is non-inferior to continuous therapy. The study by Hershman *et al*. established a link between the SWOG clinical trial numbers and Medicare claims data for a subset of the SWOG trial patients in order to analyze the incidence of specific adverse events during the trial period. In total, 636 patients were included in this analysis, and 76% were over the age of 65 when they initially enrolled in the SWOG trial. The authors studied the overall incidence of adverse events and compared the incidences for patients who had received continuous versus intermittent ADT. Baseline patient characteristics were similar between the two groups. They found that the most common adverse events were endocrine (41%), particularly hypercholesterolemia, which occurred in 31% of the patients studied. Adverse events related to bone health were also common; 19% of patients were diagnosed with osteoporosis, and 14% were diagnosed with a fracture. Ischemic and thrombotic events occurred in 27% of patients, and 10% of patients had Medicare claims related to ischemic heart disease. The authors had hypothesized that the rate of adverse events would be higher in the continuous ADT group than the intermittent group, but the results did not support this hypothesis. The cumulative 10-year incidence of a thrombotic or ischemic event was surprisingly higher for the intermittent therapy group (33%) than for the continuous therapy group (24%) (HR = 0.69, *p* = 0.02). The cumulative 10-year incidence of ischemic cardiac disease was also higher in the intermittent therapy group (12%) than in the continuous therapy group (7%) (HR = 0.55, *p* = 0.05). There was no significant difference in the incidence of other adverse events between the two groups. This trial has some notable limitations, including the fact that using coding from Medicare claims data is associated with room for error when assessing which patients developed the adverse events that were studied. However, it does provide valuable information regarding adverse events associated with ADT and the incidence of such events in patients on continuous versus intermittent ADT.

An interesting study of “nocebo effects” in elders was also published last year ^10^. A nocebo effect is a negative adverse effect from an inert substance, such as a placebo pill given in a randomized chemotherapy clinical trial. This study looked at two large cancer cooperative group studies which included a placebo arm and was stratified by age (less than or more than 65 years). The interesting finding of this study was that, even in the placebo arms, over 5,000 adverse events were reported across 446 patients. Notably, there were no differences in nocebo effect by age. The high rate of adverse events reported in the placebo arms is likely owing in part to the fact that, at baseline, patients with cancer often have significant morbidity which can result in a high rate of adverse events. Elderly patients especially often have significant comorbidities which may contribute to perceived adverse events related to treatment. Controlling for this fact is important in clinical trial design of novel therapeutics. In addition, the negative adverse events may be due to patient expectations that a treatment is likely to cause harm. Adequate trial design is also important to limit the potential for the placebo and nocebo effects to confound result interpretation.

## Geriatric fitness assessments and age as predictors of outcomes

The next study, by Aparicio *et al*. ^11^, attempted to use geriatric evaluations as predictors of response to chemotherapy and OS. This study randomly assigned 282 patients with unresectable metastatic colorectal cancer over the age of 75 years to either single-agent 5-fluoruracil or combination therapy with irinotecan plus 5-fluorouracil. The participants also had the option of completing a baseline geriatric assessment, which included the Charlson Comorbidity Index, Mini-Mental State Examination of cognition, Quality of Life, Geriatric Depression Scale, and Instrumental Activities of Daily Living (IADL). About half of the enrolled patients completed these assessments. Multivariate analysis revealed that no geriatric evaluation or measure was predictive for objective response rates to chemotherapy or PFS. However, patients who had no impairments in their IADLs were found to have improved OS when compared with patients with impaired IADLs. The addition of irinotecan did not improve PFS and had no effect on OS but did increase the objective response rate.

In contrast to Aparicio *et al*., Ribi *et al*. ^12^ were able to demonstrate in their work that a cancer-specific geriatric assessment was predictive for toxicity in older adults who received rituximab, bendamustine, and lenalidomide for aggressive B-cell lymphoma. Specifically, older adults with Vulnerable Elders Survey-13 (VES-13) scores of greater than 2 were more likely to have toxicity, show poor response to chemotherapy, and die during treatment. The VES-13 specifically looks at functional status and is scored from 0 to 10. Other tests, including Charlson Comorbidity Score, Geriatric Depression Scale, Mini-Nutritional Assessment, Mini-Cog ^©^, and the Modified Medical Outcomes Study (MOS) Social Support Survey, did not predict for outcome. The older adults enrolled in this trial were otherwise deemed ineligible for treatment with standard anthracycline-based chemotherapy. This study, having enrolled only 57 patients, is small. However, it does add to the data which suggest that functional status is the most crucial geriatric domain and that geriatric assessments are most useful to predict toxicity to chemotherapy over other cancer-related outcomes.

With regard to using age as a predictor of outcome, Bishop *et al*. analyzed a population of women older than 70 years with endometrial cancer ^13^. This study was a post-hoc analysis of the Gynecology Oncology Group (GOG) LAP2 trial, which randomly assigned patients to laparotomy versus laparoscopy for surgical staging of endometrial cancer. Older patients had worse PFS and OS and generally more aggressive cancers. These findings were observed despite similar rates of adjuvant therapy. This study also showed that the GOG 99 high- to intermediate-risk criteria for endometrial cancer, which include a point for being over 70, were not predictive in older adults unless all three uterine factors, rather than the usual two, were included.

Geriatric fitness assessments have also been shown to have value in the setting of advanced NSCLC. The 2011 trial by Quoix *et al*. ^2^ found that a baseline ADL score of 6 was associated with better OS than a baseline ADL score of less than 6. However, the baseline ADL score did not provide predictive value in terms of response to chemotherapy. Another trial, conducted by Corre *et al*. ^14^ in 2016, studied the utility of a comprehensive geriatric assessment (CGA) when choosing a chemotherapy regimen for elderly patients with advanced NSCLC. Patients were 70 or older with a PS of 0 to 2. They were randomly assigned to treatment allocation on the basis of age and PS (standard arm) or on the basis of CGA (CGA arm). For the standard arm, patients received a carboplatin-based doublet for a PS of less than 2 or age of not more than 75, and they received docetaxel for a PS of 2 or age of more than 75 years. For the CGA arm, fit patients received a carboplatin-based doublet, vulnerable patients received docetaxel, and frail patients received best supportive care. The CGA included PS, ADLs, IADLs, Mini-Mental State Examination, assessment for presence of a geriatric syndrome, Charlson Comorbidity Index, and Geriatric Depression Scale. The authors found no significant difference in the rates of treatment failure-free survival or OS between the standard arm and the CGA arm. However, patients in the CGA arm did experience significantly less all-grade toxicity (85.6% versus 93.4%, respectively; *p* = 0.015) and fewer treatment failures as a result of toxicity (4.8% versus 11.8%, respectively; *p* = 0.007). These results highlight the potential utility of a CGA to help reduce the risk of therapy-associated toxicities for elderly patients with advanced NSCLC.

## Advancements in palliative and supportive care

Optimal palliative care is a paramount issue for older adults with advanced cancer. There were three trials that addressed issues of palliative and supportive care in older patients last year ^15– 17^. In the first study ^15^, the effect of antipsychotics in “terminal delirium” among palliative care patients was explored. This study was a randomized trial of oral risperidone, haloperidol, or placebo for managing symptoms of delirium among 247 patients in palliative care. Specifically, this study looked at behavioral, communication, and perceptual symptoms of delirium. Most of the enrolled patients were older (mean age of nearly 75 years) and most had cancer as the reason for enrollment in palliative care. In both the risperidone and the haloperidol arms, delirium was worse than placebo, and OS was shorter in the haloperidol arm (but not the risperidone arm). Both intervention arms also had more extrapyramidal side effects. This study definitively concludes that antipsychotic drugs should not be used to manage symptoms of delirium in patients receiving palliative care. This is a practice-changing study.

Care for medical devices is a special concern for older adults, who frequently have loss of vision and dexterity as they age. Liu *et al*. ^16^ looked at the optimal skin barrier for older adults with colostomies after surgery for colorectal cancer. Patients were randomly assigned to a standard skin barrier which was cut to the size of the ostomy versus a moldable skin barrier which was stretched to the size of the ostomy opening for their colostomy apparatus. Investigators looked at the incidence of peristomal dermatitis, patient satisfaction, and costs. There was significantly ( *p* <0.05) less dermatitis in the moldable skin barrier arm. There was no difference in cost or time, but the control arm did have to use more barrier cream to prevent leakage ( *p* <0.01). This trial supports the use of moldable ostomy skin barriers in older adults with colorectal cancer-related colostomies.

A randomized trial was published last year looking at increasing the comfort of inpatient nurses and family caregivers who are caring for older adults who are dying in the hospital ^17^. This was a cluster randomized controlled trial on inpatient geriatric wards in Belgium of an educational program around death and dying (Care Programme for the Last Days of Life, or CAREFuL) versus usual care. The CAREFuL program is geared toward geriatric health-care staff and family members of a dying patient. It is meant to facilitate higher-quality care for elderly patients who are dying in an inpatient care facility. The program involves several components. One is a care guide for health-care staff during the last days of life, including points of attention regarding specific symptoms as well as a checklist regarding how to provide the family with information about the dying process. Another component is supportive documentation which provides general information for health-care staff on how to use the care guide as well as three leaflets for family members on entering the dying phase, facilities available on the inpatient unit, and grief and bereavement. The final component is an implementation guide. There were several validated metrics that were used for results assessment in this study. One was the Comfort Assessment in Dying–End-of-Life in Dementia (CAD-EOLD) scale, which is a 14-item questionnaire in which each item is a specific symptom or sign (for example, pain and moaning) indicative of how comfortable the patient is during the dying process. Nurses and family members rated how frequently patients experienced these symptoms. From the perspective of nurses, patients in the CAREFuL program had significantly better CAD-EOLD scores than patients in the control group ( *p* <0.0001), but there was no significant difference in the CAD-EOLD scores between the two groups when assessed by family members. Another metric used in this study was a modified version of the Symptom Management Scale–End-of-Life in Dementia (SM-EOLD). This is a nine-item form in which each item corresponds to a specific symptom (for example, shortness of breath, anxiety, and agitation). Nurses and family members were asked to rate how frequently patients experienced these symptoms. There was no significant difference between the CAREFuL and control groups for this scale when assessed by nurses or family members. Another metric was the End-of-Life in Dementia Satisfaction with Care Scale, which is a 10-item scale regarding family members’ satisfaction with the care that the patient received at the end of life. Scores on this scale were significantly worse for the CAREFuL group than the control group. This finding regarding family members’ satisfaction with care deserves further exploration, and one cannot advocate for the broad use of the CAREFuL program at this time.

In conclusion, there were many interesting and important studies published this past year in geriatric oncology. We learned better ways to support patients through cancer treatments. We refined our understanding of the utility of geriatric assessments in patients with cancer. We developed the body of information about which chemotherapy agents are safe and effective in older adults (and which are not) as well as late effects of cancer-directed therapy in older adults. We also expanded our understanding of good palliative and supportive care. Future work should further explore appropriate chemotherapy options, and more trials should include “real” older adults with multiple comorbid conditions, many concurrent medications, and poorer PS. Such inclusion will allow us to better apply study results to our day-to-day care of older patients, many of whom do not currently qualify for most clinical trials in oncology.
